# Time to publication among completed diagnostic accuracy studies: associated with reported accuracy estimates

**DOI:** 10.1186/s12874-016-0177-4

**Published:** 2016-06-06

**Authors:** Daniël A. Korevaar, Nick van Es, Aeilko H. Zwinderman, Jérémie F. Cohen, Patrick M. M. Bossuyt

**Affiliations:** Department of Clinical Epidemiology, Biostatistics and Bioinformatics, Academic Medical Center, University of Amsterdam, Meibergdreef 9, 1105 AZ Amsterdam, The Netherlands; Department of Vascular Medicine, Academic Medical Center, University of Amsterdam, Amsterdam, The Netherlands; Inserm U1153, Center for Epidemiology and Statistics Sorbonne Paris Cité, Paris Descartes University, Paris, France

**Keywords:** Reporting bias, Time lag bias, Diagnostic accuracy, Sensitivity and specificity, Research waste, Trial registration

## Abstract

**Background:**

Previous evaluations have documented that studies evaluating the effectiveness of therapeutic interventions are not always reported, and that those with statistically significant results are published more rapidly than those without. This can lead to reporting bias in systematic reviews and other literature syntheses. We evaluated whether diagnostic accuracy studies that report promising results about the performance of medical tests are also published more rapidly.

**Methods:**

We obtained all primary diagnostic accuracy studies included in meta-analyses of Medline-indexed systematic reviews that were published between September 2011 and January 2012. For each primary study, we extracted estimates of diagnostic accuracy (sensitivity, specificity, Youden’s index), the completion date of participant recruitment, and the publication date. We calculated the time from completion to publication and assessed associations with reported accuracy estimates.

**Results:**

Forty-nine systematic reviews were identified, containing 92 meta-analyses and 924 unique primary studies, of which 756 could be included. Study completion dates were missing for 285 (38 %) of these. Median time from completion to publication in the remaining 471 studies was 24 months (IQR 16 to 35). Primary studies that reported higher estimates of sensitivity (Spearman’s rho = −0.14; *p =* 0.003), specificity (rho = −0.17; *p <* 0.001), and Youden’s index (rho = −0.22; *p <* 0.001) had significantly shorter times to publication. When comparing time to publication in studies reporting accuracy estimates above versus below the median, the median number of months was 23 versus 25 for sensitivity (*p =* 0.046), 22 versus 27 for specificity (*p =* 0.001), and 22 versus 27 for Youden’s index (*p <* 0.001). These differential time lags remained significant in multivariable Cox regression analyses with adjustment for other study characteristics, with hazard ratios of publication of 1.06 (95 % CI 1.02 to 1.11; *p =* 0.007) for logit-transformed estimates of sensitivity, 1.09 (95 % CI 1.04 to 1.14; *p <* 0.001) for logit-transformed estimates of specificity, and 1.09 (95 % CI 1.03 to 1.14; *p =* 0.001) for logit-transformed estimates of Youden’s index.

**Conclusions:**

Time to publication was significantly shorter for studies reporting higher estimates of diagnostic accuracy compared to those reporting lower estimates. This suggests that searching and analyzing the published literature, rather than all completed studies, can produce a biased view of the performance of medical tests.

**Electronic supplementary material:**

The online version of this article (doi:10.1186/s12874-016-0177-4) contains supplementary material, which is available to authorized users.

## Background

Many completed biomedical studies take years to get published, if they get published at all [1, 2]. Over the past decades, there have been increasing concerns about the resulting bias for those relying on a synthesis of the available literature in getting summary estimates of the effectiveness of therapeutic interventions [3–5]. There is now overwhelming evidence that studies with statistically non-significant results are less likely to result in a publication in a peer-reviewed journal than those with statistically significant results [1, 2, 6, 7]. Evaluations have also shown that it takes more time before negative studies are published [8–11]. There are multiple reasons for non- or delayed publication of studies with non-significant findings. Researchers, anticipating low scientific impact, may be reluctant to write and submit the study report; journals, foreseeing low citation rates, may be less interested in publishing them [12, 13].

Diagnostic accuracy studies evaluate the ability of medical tests to differentiate between patients with and without a target condition. It is unknown whether such studies are also susceptible to differential publication processes, with studies that document disappointing results about a test’s performance being less likely to be published in full, or published later, compared to studies reporting more promising findings [14–17]. In itself, statistical significance is unlikely to be a major determinant of time to publication among diagnostic accuracy studies; these studies typically present results only in terms of estimates of sensitivity and specificity, and most do not have specific hypothesis tests and accompanying *p*-values [18–21]. It is possible, however, that the sheer magnitude of the reported accuracy estimates can be seen as a measure of the favorability of the study findings, and that studies reporting higher accuracy estimates are published sooner than studies reporting lower accuracy estimates.

The objective of this study was to evaluate whether reported accuracy estimates were associated with time to publication among published diagnostic accuracy studies.

## Methods

### Selection of diagnostic accuracy studies

We relied on a set of 114 Medline-indexed systematic reviews of diagnostic accuracy studies, published in English between September 2011 and January 2012. These reviews were identified in a previous meta-epidemiological project from our research group. The search and selection process have been described in full elsewhere [21].

These systematic reviews were included in the current evaluation if they contained one or more meta-analyses and provided 2x2 tables for the primary studies included in these meta-analyses, describing the number of true and false positive and negative results for the diagnostic test under investigation. For each primary study included in the meta-analyses we then obtained the full study report or, if not available, the abstract.

### Data extraction

For each primary study, two investigators (DAK, JFC) independently extracted the test under evaluation, and the 2x2 tables reported in the meta-analyses. These investigators also independently identified the publication date.

For Medline-indexed studies, the date on which the citation was added to the PubMed database was used as the publication date. For studies not indexed in Medline, we tried to obtain the publication date through Google Scholar, the journal website, or the full study report. Primary studies for which no publication date could be identified were excluded from further analysis, as were conference abstracts.

One investigator (DAK or NvE) then extracted additional data from the articles in which the primary studies were reported. A random 10 % of this data extraction was independently verified by the other investigator; discrepancies occurred in 3 out of 632 (0.5 %) verified characteristics.

We extracted the start date and completion date of participant recruitment, the date of first submission to the publishing journal, and the date the study was accepted for publication. If only the months but not the exact dates of participant recruitment were provided, start dates were rounded to the first day of that month, whereas completion dates were rounded to the last day of that month. If only years of participant recruitment were provided, start dates were rounded to January 1^st^ of the starting year, and completion dates to December 31^st^ of the completion year.

We also extracted the journal in which the study was published and corresponding 2014 impact factor (through Web of Knowledge), number of authors, country of first author, and type of data collection (prospective/retrospective). Data extraction from study reports published in non-English language was performed with the help of native speakers, or using Google Translate. Any disagreements in the data extraction process were resolved through discussion.

### Data analysis

For each included primary study, we recalculated estimates of sensitivity and specificity from the extracted 2x2 tables. Because tests may have a high sensitivity but a low specificity, or the other way around, we also calculated Youden’s index (sensitivity *plus* specificity *minus* 1). This is a single measure of diagnostic accuracy that takes the whole 2x2 table into account [22]. If multiple 2x2 tables were available for one primary study—which could happen, for example, because multiple tests had been evaluated—the highest reported estimates of sensitivity, specificity and Youden’s index were used in the analyses.

Our analysis focused on time from completion to publication, defined as the time interval between the completion date and the publication date. This was further subdivided in time from completion to submission, and time from submission to publication.

We calculated Spearman’s rho correlation coefficients between accuracy estimates and time from completion to publication; a negative correlation coefficient meaning that studies reporting higher estimates had shorter times to publication. To further quantify potential delays, estimates of sensitivity, specificity and Youden’s index were then dichotomized by a median split, and median times from completion to publication were compared using Mann–Whitney U tests. To explore more specifically in which phase potential delays in time from completion to publication occurred, this analysis was repeated for time from completion to submission, and for time from submission to publication. Studies with partially missing dates were only excluded from the analyses for that specific time interval.

We performed multivariable Cox proportional hazards regression analysis to evaluate the unconditional and conditional effect of accuracy estimates on the hazard of publication, adjusting for year of publication, journal impact factor (≥4 versus <4 or not available), number of authors, continent (Europe, North America or Oceania versus Asia, Africa or South America), type of test (imaging versus other), type of data collection (prospective versus retrospective or not reported), study duration (time interval between the start date and completion date), and number of participants in the 2x2 table, adding a frailty term per meta-analysis to account for systematic differences in time to publication between meta-analyses. In this analysis, accuracy estimates were logit transformed, where a correction was applied for accuracy estimates of exactly 0 or 1; these were considered to be 0.001 or 0.999. Other continuous variables were not transformed before adding them to the models. This analysis was also repeated for time from completion to submission, and time from submission to publication.

### Sensitivity analysis

We performed sensitivity analysis by excluding primary studies that only provided the year, but not the month or exact date of completion of participant recruitment, as these calculations of time from completion to publication were likely to be less accurate. We also performed sensitivity analysis by excluding studies that did not provide both a completion date and a submission date, thereby restricting the analysis to studies for which we had both time from completion to publication, time from completion to submission, and time from submission to publication (complete case analysis). Data were analyzed in SPSS v.22 and R v.3.0.3 [23, 24].

## Results

### Selection of diagnostic accuracy studies

Details on the selection of studies and a list of included systematic reviews are provided in Additional files 1 and 2, respectively. In total, 49 systematic reviews could be included in the current evaluation, containing 92 meta-analyses. Together, these meta-analyses contained 924 unique primary diagnostic accuracy studies. Of these, 168 (18 %) had to be excluded because no publication date could be obtained (*n =* 163), because they were conference abstracts (*n =* 4), or because they had been retracted (*n =* 1).

The remaining 756 primary diagnostic accuracy studies were included, corresponding to 1,088 2x2 tables, as some studies were included in multiple meta-analyses. A full study report could be obtained for 751 of these; for the other 5 studies, data extraction was performed using the abstract only.

### Study characteristics

Nineteen diagnostic accuracy studies (3 %) were published before 1990; 133 (18 %) between 1990 and 2000; 527 (70 %) between 2000 and 2010; and 77 (10 %) between 2010 and 2012. They were published in 322 different journals, most frequently in *European Journal of Nuclear Medicine and Molecular Imaging* (*n =* 30; 4 %), *Radiology* (*n =* 27; 4 %), *American Journal of Roentgenology* (*n =* 20; 3 %), and *Journal of Clinical Microbiology* (*n =* 20; 3 %). The median impact factor was 3.1 (inter quartile range [IQR] 2.0 to 5.4).

Study reports were in 10 different languages, most frequently in English (*n =* 726; 96 %). The median number of authors was 6 (IQR 5 to 8). First authors were from 64 different countries, most frequently USA (*n =* 153; 20 %), Germany (*n =* 60; 8 %), and Japan (*n =* 54; 7 %).

The type of test under investigation was an imaging test for 387 studies (51 %) and another type of test for 369 studies (49 %). Data collection was prospective in 307 studies (41 %), retrospective in 125 studies (17 %), and not reported in 324 studies (43 %). The median study duration was 22 months (IQR 12 to 37), with a median number of participants of 100 (IQR 49 to 255). The median accuracy estimates were 0.875 (IQR 0.73–0.97) for sensitivity, 0.899 (0.76–0.97) for specificity, and 0.684 (0.45–0.83) for Youden’s index.

### Time to publication: association with reported estimates of diagnostic accuracy

**Fig. 1:**
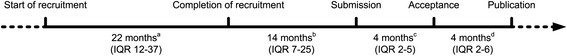
Median times between study stages. Median times missing for: ^a^246; ^b^426; ^c^275; and ^d^192 of 756 included studies

Of the included primary studies, 520 (69 %) reported a submission date, 564 (75 %) an acceptance date, 474 (63 %) a start date, and 471 (62 %) a completion date. Median times between study stages are summarized in Fig. 1.

**Fig. 2:**
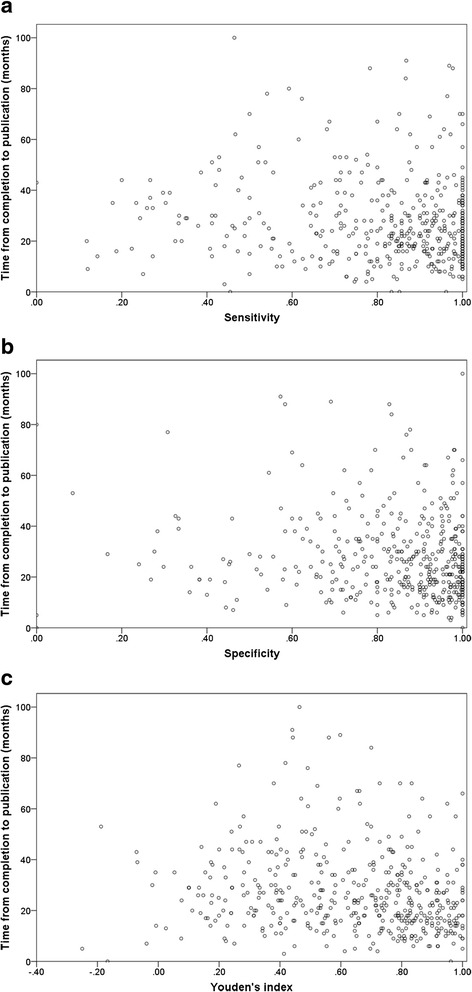
Correlations between reported estimates of diagnostic accuracy and time from completion to publication. **a** Sensitivity. **b** Specificity. **c** Youden’s index. Each dot represents one diagnostic accuracy study

The median time from completion to publication (available for 471 studies) was 24 months (IQR 16 to 35). Sensitivity (rho = −0.14; *p =* 0.003), specificity (rho = −0.17; *p <* 0.001), and Youden’s index (rho = −0.22; *p <* 0.001) were each negatively correlated with time from completion to publication (Fig. 2).

**Fig. 3:**
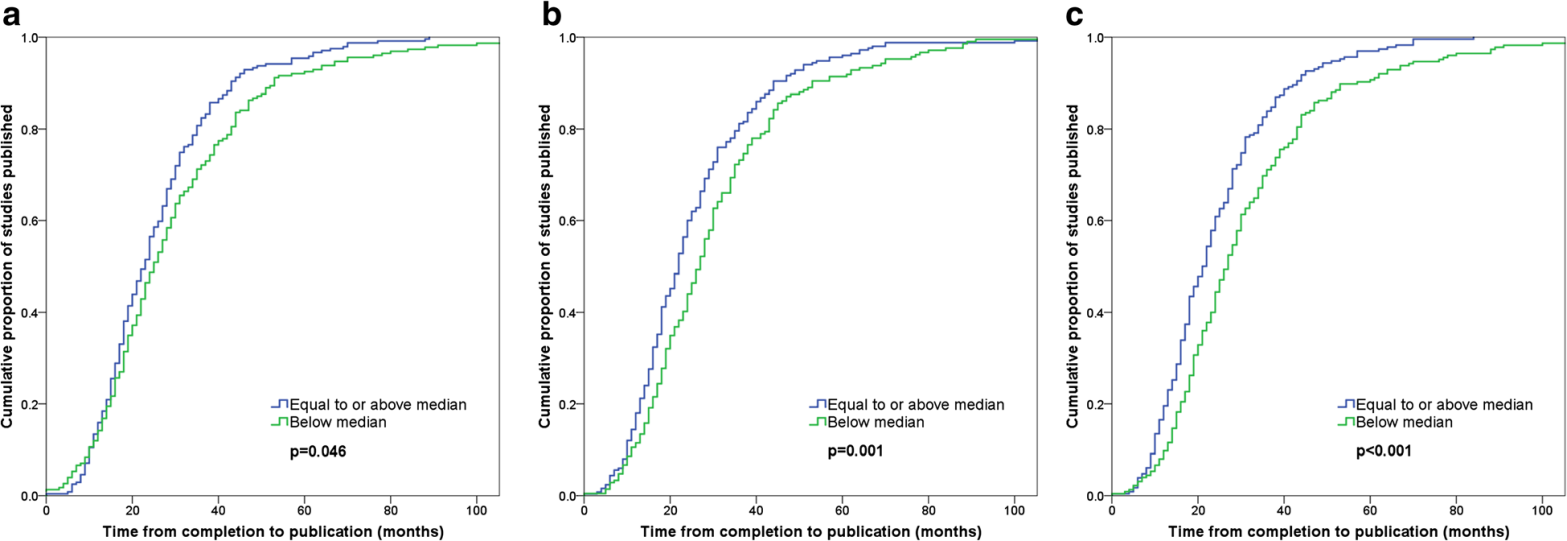
Time from completion to publication. **a** Sensitivity. **b** Specificity. **c** Youden’s index

When comparing time from completion to publication in studies reporting accuracy estimates above versus below the median, the median number of months was 23 versus 25 for sensitivity (*p =* 0.046), 22 versus 27 for specificity (*p =* 0.001), and 22 versus 27 for Youden’s index (*p <* 0.001) (Table 1; Fig. 3). Median time from completion to publication stratified by other categories of study characteristics is provided in Table 2.

**Table 1:** Time to publication: association with dichotomized accuracy estimates

		Time from completion to publication^a^			Time from completion to submission^b^			Time from submission to publication^c^		
		Studies n (%)	Months Median (IQR)	*p*-value	Studies n (%)	Months Median (IQR)	*p*-value	Studies n (%)	Days Median (IQR)	*p*-value
Overall		471 (100 %)	24 (16–35)		330 (100 %)	14 (7–25)		520 (100 %)	238 (177–329)	
Sensitivity^d^										
	<0.875	226 (48 %)	25 (16–39)	0.046	149 (45 %)	16 (7–30)	0.037	225 (43 %)	240 (176–324)	0.755
	≥0.875	239 (51 %)	23 (15–32)		179 (54 %)	13 (7–22)		293 (56 %)	238 (183–335)	
Specificity^e^										
	<0.899	209 (44 %)	27 (18–38)	0.001	152 (46 %)	17 (9–30)	0.001	262 (50 %)	238 (176–317)	0.494
	≥0.899	250 (53 %)	22 (15–31)		173 (52 %)	12 (6–22)		252 (48 %)	238 (180–332)	
Youden’s index^f^										
	<0.684	225 (48 %)	27 (18–39)	<0.001	157 (48 %)	17 (10–30)	<0.001	245 (47 %)	235 (176–321)	0.251
	≥0.684	230 (49 %)	22 (14–31)		166 (50 %)	11 (6–21)		267 (51 %)	244 (180–332)	

Median times missing for: ^a^285, ^b^426, and ^c^236 of 756 included studies

^d^Sensitivity, ^e^Specificity, and ^f^Youden’s index missing for 7, 14 and 18 of 756 included studies, respectively

**Table 2:** Time from completion to publication: association with other study characteristics

		Studies n (%)	Months Median (IQR)
Overall^a^		471 (100 %)	24 (16–35)
Year of publication			
	<1990	10 (2 %)	26 (17–53)
	1990–1994	15 (3 %)	18 (14–38)
	1995–1999	44 (9 %)	23 (15–36)
	2000–2004	99 (21 %)	26 (18–37)
	2005–2009	243 (52 %)	23 (16–35)
	≥2010	60 (13 %)	22 (15–35)
Journal impact factor			
	<4 or not available	312 (66 %)	25 (16–37)
	4–9	129 (27 %)	22 (15–33)
	≥10	30 (6 %)	22 (16–34)
Number of authors			
	<6	170 (36 %)	23 (14–38)
	≥6	301 (64 %)	24 (17–34)
Continent of first author			
	Africa	32 (7 %)	31 (20–48)
	Asia	125 (27 %)	20 (13–29)
	Europe	183 (39 %)	24 (17–35)
	North America	116 (25 %)	26 (16–39)
	Oceania	10 (2 %)	28 (21–44)
	South America	5 (1 %)	19 (14–50)
Type of test			
	Imaging	229 (49 %)	24 (16–34)
	Other	242 (51 %)	24 (16–37)
Type of data collection			
	Prospective	192 (41 %)	24 (17–34)
	Retrospective	96 (20 %)	25 (15–36)
	Not reported	183 (39 %)	24 (15–38)
Study duration^b^			
	<13 months	115 (24 %)	24 (15–34)
	13–24 months	139 (30 %)	25 (19–35)
	≥25 months	216 (46 %)	24 (14–37)
Number of participants			
	<100	208 (44 %)	24 (15–35)
	100–999	232 (49 %)	24 (16–35)
	≥1000	31 (7 %)	27 (20–39)

^a^Time from completion to publication missing for 285 of 756 included studies

^b^Study duration missing for 1 of 471 studies included in this analysis

These differential time lags remained significant in multivariable Cox regression analyses, with hazard ratios of publication of 1.06 (95 % confidence interval [CI] 1.02 to 1.11; *p =* 0.007) for logit-transformed estimates of sensitivity, 1.09 (95 % CI 1.04 to 1.14; *p <* 0.001) for logit-transformed estimates of specificity, and 1.09 (95 % CI 1.03 to 1.14; *p =* 0.001) for logit-transformed estimates of Youden’s index (Table 3).

**Table 3:** Time from completion to publication: multivariable Cox regression analyses

		Hazard ratio (95 % CI)^a^	*p*-value
Model 1: Sensitivity (*n =* 464)			
Sensitivity (logit transformed)		1.06 (1.02–1.11)	0.007
Year of publication (per 5 years)		0.98 (0.88–1.08)	0.660
Journal impact factor			
	≥4	1.24 (0.99–1.55)	0.064
	<4 or not available	1	
Number of authors		0.99 (0.96–1.02)	0.550
Continent of first author			
	Europe, North America or Oceania	0.69 (0.55–0.87)	0.002
	Africa, Asia or South America	1	
Type of test			
	Imaging	1.15 (0.88–1.50)	0.300
	Other	1	
Type of data collection			
	Prospective	1.19 (0.96–1.48)	0.120
	Retrospective or not reported	1	
Study duration (per year)^b^		1.00 (0.98–1.03)	0.820
Number of participants (per 1000)		1.01 (0.99–1.03)	0.430
Model 2: Specificity (*n =* 458)			
Specificity (logit transformed)		1.09 (1.04–1.14)	<0.001
Year of publication (per 5 years)		1.01 (0.91–1.11)	0.910
Journal impact factor			
	≥4	1.34 (1.07–1.67)	0.011
	<4 or not available	1	
Number of authors		0.99 (0.96–1.02)	0.360
Continent of first author			
	Europe, North America or Oceania	0.72 (0.57–0.90)	0.003
	Africa, Asia or South America	1	
Type of test			
	Imaging	1.15 (0.90–1.46)	0.260
	Other	1	
Type of data collection			
	Prospective	1.23 (1.00–1.52)	0.050
	Retrospective or not reported	1	
Study duration (per year)^b^		1.00 (0.98–1.02)	0.980
Number of participants (per 1000)		1.01 (0.99–1.03)	0.540
Model 3: Youden’s index (*n =* 454)			
Youden’s index (logit transformed)^g^		1.09 (1.03–1.14)	0.001
Year of publication (per 5 years)		0.98 (0.89–1.09)	0.730
Journal impact factor			
	≥4	1.28 (1.02–1.61)	0.031
	<4 or not available	1	
Number of authors		0.98 (0.95–1.01)	0.280
Continent of first author			
	Europe, North America or Oceania	0.69 (0.55–0.87)	0.002
	Africa, Asia or South America	1	
Type of test			
	Imaging	1.16 (0.90–1.51)	0.250
	Other	1	
Type of data collection			
	Prospective	1.24 (1.00–1.54)	0.052
	Retrospective or not reported	1	
Study duration (per year)^b^		1.00 (0.98–1.02)	0.910
Number of participants (per 1000)		1.01 (0.99–1.03)	0.460

^a^Frailty term added per meta-analysis to account for systematic differences in time from completion to publication between meta-analyses; variance of frailty terms was: model 1 = 0.103; model 2 = 0.064; model 3 = 0.094

^b^One study was excluded from the Cox regression analysis because of a missing study duration

When subdividing time from completion to publication, we observed significant associations between accuracy estimates and time from completion to submission (available for 330 studies), but not between accuracy estimates and time from submission to publication (available for 520 studies) (Table 1, with multivariable Cox regression analyses in Additional files 3 and 4).

### Sensitivity analysis

The sign and significance of the association between estimates of diagnostic accuracy and time from completion to publication, time from completion to submission, and time from submission to publication remained the same when excluding studies that only reported the year of completion of participant recruitment but not the month or exact date, and when excluding studies that did not report both a completion date and a submission date (Additional file 5).

## Discussion

In a large sample of published diagnostic accuracy studies, we found that it took authors on average two years to publish study findings after completing participant recruitment. Time from completion to publication was significantly shorter for studies reporting higher estimates of diagnostic accuracy compared to those reporting lower estimates, a delay that could not be attributed to differences in speed of processing within the journals that eventually published the studies.

Some elements deserve consideration. Many reports of diagnostic accuracy studies contain multiple accuracy outcomes, for example, for different tests, target conditions, and subgroups. We only obtained the 2x2 tables that were used in the selected meta-analyses, but the primary studies may have focused on other accuracy outcomes as well. Whenever a study reported multiple 2x2 tables, we selected the highest accuracy estimates in our analysis, because in our personal experience authors have a tendency to emphasize these in their conclusions. However, whether the highest accuracy estimates in a study are indeed the ones that drive time to publication is unknown. In our analysis, we focused on dichotomized accuracy estimates, as this allowed us to provide a straightforward quantification of the delays that can be anticipated in the publication of results that are relatively disappointing in diagnostic research. We acknowledge that a dichotomization in terms of a median-split is arbitrary, and that this may not reflect the difference between statistically significant and non-significant results based on the *p*-value.

Although the STAndards for Reporting Diagnostic accuracy (STARD) statement invites authors to report start and completion dates of participant recruitment [25], these were not provided by more than one-third of the studies. As a consequence, we could not include these studies in our analyses of time from completion to publication. This obviously limited the precision of our findings, but we do not know whether the included sample is a biased one. We included eight additional variables in our Cox regression analyses. It is conceivable that there are other unmeasured confounders in the association between accuracy estimates and time to publication as well. Especially several study characteristics that are associated with study quality and risk of bias, such as blinding of test readers and quality of the reference standard, may be relevant in this respect. We did not include these elements because they are often not reported, and the extent to which they induce bias varies substantially depending on the type of test under investigation and the clinical area in which the test is applied [26, 27]. However, we believe that excluding these is more likely to have led to under- rather than overestimations of the associations identified in this study: poor study quality generally leads to inflated accuracy estimates, but will probably also increase time to publication as a result of critical peer-reviewers and more journal rejections.

Several previous evaluations found a comparable differential time lag among studies of therapeutic interventions. A Cochrane review that aimed to document the association between statistically significant results and time to publication included two of such evaluations, together analyzing the fate of 196 initiated clinical trials [8–10]. On average, trials with significant results were published about 2 to 3 years earlier than those with non-significant results. In a similar, more recent evaluation of 785 initiated clinical trials, the estimated median time from completion to publication was 2.1 years for those with significant results, and 3.2 years for those that with non-significant ones [11]. A differential time lag was not identified in another evaluation of 1,336 published clinical trials: both those with significant and non-significant outcomes had a median time to publication of 21 months [28].

Similar evaluations are scarce for diagnostic accuracy studies, and so far limited to abstracts presented at scientific conferences in specific fields of research. In contrast to our findings, no systematic bias could be identified in these previous assessments. One study found a median time from presentation to full publication of 16 months for 160 conference abstracts of diagnostic accuracy studies in stroke research, but the hazard of full publication was not associated with reported estimates of Youden’s index [15]. We recently found that the median time from presentation to publication was 17 months among 399 conference abstracts of diagnostic accuracy studies in ophthalmology research; there also, the hazard of publication was not associated with reported estimates of sensitivity and specificity (*manuscript submitted for publication*).

In the current evaluation, on average, it took two months less to publish studies with a sensitivity above the median, five months less to publish studies with a specificity above the median, and five months less to publish studies with a Youden’s index above the median, compared to studies reporting estimates of these accuracy estimates below the median. Although these time lags can be considered as relatively minor, the potential implications, although difficult to overlook, may be worrisome for multiple reasons.

We believe that the observed differential time lag may reflect a larger underlying problem. A study’s chances to reach full publication are likely to fade over time and with every rejection by a journal. This may lead to failure to publish the study and, consequently, to publication bias, since the study will be missing from the evidence base to those relying on databases of published articles [3]. Although there is strong evidence of such bias in syntheses of studies of therapeutic interventions, this topic has been insufficiently investigated for diagnostic accuracy studies [14–17].

Even if studies with less favorable results are eventually published, a delay in their publication and associated dissemination can lead to misleading results in systematic reviews. Reporting bias may occur when literature reviewers want to synthesize the available evidence but cannot account for unfavorable results that take substantially longer to get published [8]. To assess time trends in published accuracy estimates, we recently applied cumulative meta-analysis to the same set of systematic reviews as used in the current evaluation [29]. Among 48 meta-analyses included, a total of 12 statistically significant time trends in sensitivity or specificity were identified. The majority of these time trends, 8 out of 12 (67 %), were negative, which may indicate that studies that are published earlier sometimes tend to overestimate the accuracy of a test. This may be partially explained by a time lag in the publication of studies that report lower accuracy estimates, as identified in the current evaluation.

The delay in publishing studies with lower accuracy estimates could be attributed to study authors, who may be less motivated to write and submit corresponding study reports, to peer reviewers, who may be more critical towards and less supportive of studies with unfavorable results, or to journal editors, who may be less willing to publish studies reporting disappointing performance of new and existing tests [12, 13]. In our evaluation, we did observe a differential time lag from study completion to submission, but not from submission to publication, indicating that delayed publication of studies with lower accuracy estimates was not caused by the journal that eventually published the study report. This suggests two alternative explanations for the delay. One is that authors were less effective, maybe even reluctant, in finalizing and submitting their study report. Another explanation is that the manuscript was not accepted by the journals where authors initially submitted the report to, and this could have been caused, in part, by the less positive findings.

In the multivariable Cox regression analysis for Youden’s index, two additional variables were also significantly associated with time from completion to publication. Studies published in journals with a higher impact factor were published more rapidly. An explanation could be that authors first submit their study to higher impact factor journals, going down after each rejection, which would then delay publication. When pooling studies from Africa, Asia and South America, these were published more rapidly than those from Europe, North America and Oceania. Little is known about geographical differences in quality and rigorousness of the editorial and peer-review processes of biomedical journals.

The findings of this study are relevant for scholars that want to arrive at a synthesis of the available evidence through a search of the literature, and for patients, clinicians, policy makers and funders, that may rely on these literature syntheses. They should be fully aware that it is very well possible that not all completed diagnostic accuracy studies have been published at the time of the evaluation, and that this could introduce reporting bias. Such bias is likely to be more pronounced if only few published studies are available. As recommended in current guidelines [17], additional efforts should be made to identify and include unpublished studies in systematic reviews, as this will strengthen the validity and improve the precision and applicability of the results.

Concerns about reporting bias were one factor that prompted the implementation of trial registration policies [30]. The International Committee of Journal Editors now only considers trials for publication if they were registered in a publically accessible trial registry before study start [31]. Unfortunately, currently only 15 % of published diagnostic accuracy studies are being registered [32]. Over the past years, evidence that many diagnostic accuracy studies remain unpublished has accumulated [14–17], making a strong case for a firmer implementation of registration policies for these studies [33–36]. The fact that the current evaluation suggests that there may also be bias in the process of publishing diagnostic accuracy studies further amplifies this message.

Registration of diagnostic accuracy studies would enable the identification of all relevant studies in a timely manner, not only those that have been published. Funders, governmental organizations and academic institutions could also require the publication of results within a year after study completion, as currently required by the Food and Drug Administration (FDA) for certain trials [37]. In an era of transparency and open access, stakeholders involved in biomedical research should make efforts to ensure that study results become available in a timely manner; this should apply to all studies, not just those presenting promising, optimistic and fascinating results [5, 38].

## Conclusions

Time to publication was significantly shorter for studies reporting higher estimates of diagnostic accuracy compared to those reporting lower estimates. This suggests that searching the published literature, rather than all completed studies, can produce a biased view of the performance of medical tests.

## Abbreviations

CI, confidence interval; IQR, inter quartile range

## Additional files

Additional file 1: Selection of studies. (DOC 176 kb) Additional file 2: List of included systematic reviews (*n =* 49). (DOC 38 kb) Additional file 3: Time from completion to submission: multivariable Cox regression analyses. (DOC 77 kb) Additional file 4: Time from submission to publication: multivariable Cox regression analyses. (DOC 77 kb) Additional file 5: Time to publication: sensitivity analyses. (DOC 62 kb)
