# Supplementary material for: Time to publication among completed diagnostic accuracy studies: associated with reported accuracy estimates
Source: BMC Med Res Methodol. 2016 Jun 6;16:68. doi: 10.1186/s12874-016-0177-4 (PMC4896017; doi:10.1186/s12874-016-0177-4)
Supplement: Additional file 1: — Selection of studies. (DOC 176 kb) [file 12874_2016_177_MOESM1_ESM.doc]

**Additional File 1:** Selection of studies.
