# Supplementary material for: Time to publication among completed diagnostic accuracy studies: associated with reported accuracy estimates
Source: BMC Med Res Methodol. 2016 Jun 6;16:68. doi: 10.1186/s12874-016-0177-4 (PMC4896017; doi:10.1186/s12874-016-0177-4)
Supplement: Additional file 2: — List of included systematic reviews (n = 49). (DOC 38 kb) [file 12874_2016_177_MOESM2_ESM.doc]

**Additional File 2:** List of included systematic reviews (n=49).

1. Barger RL, Jr., Nandalur KR. Diagnostic performance of dual-time 18F-FDG PET in the diagnosis of pulmonary nodules: a meta-analysis. *Academic radiology.* 2012;19(2):153-158.

2. Besson FL, Parienti JJ, Bienvenu B, et al. Diagnostic performance of (1)(8)F-fluorodeoxyglucose positron emission tomography in giant cell arteritis: a systematic review and meta-analysis. *European journal of nuclear medicine and molecular imaging.* 2011;38(9):1764-1772.

3. Brush J, Boyd K, Chappell F, et al. The value of FDG positron emission tomography/computerised tomography (PET/CT) in pre-operative staging of colorectal cancer: a systematic review and economic evaluation. *Health technology assessment.* 2011;15(35):1-192, iii-iv.

4. Cheng X, Li Y, Liu B, Xu Z, Bao L, Wang J. 18F-FDG PET/CT and PET for evaluation of pathological response to neoadjuvant chemotherapy in breast cancer: a meta-analysis. *Acta radiologica.* 2012;53(6):615-627.

5. Ding W, Shen Y, Yang J, He X, Zhang M. Diagnosis of pneumothorax by radiography and ultrasonography: a meta-analysis. *Chest.* 2011;140(4):859-866.

6. Dong MJ, Zhao K, Liu ZF, Wang GL, Yang SY, Zhou GJ. A meta-analysis of the value of fluorodeoxyglucose-PET/PET-CT in the evaluation of fever of unknown origin. *European journal of radiology.* 2011;80(3):834-844.

7. Gao F, Ren L, Zhang CQ, Mu FY, You YQ, Liu YH. Diagnostic value of anti-cyclic citrullinated peptide antibody for rheumatoid arthritis in a Chinese population: a meta-analysis. *Rheumatol Int.* 2012;32(10):3201-3218.

8. Gargiulo P, Petretta M, Bruzzese D, et al. Myocardial perfusion scintigraphy and echocardiography for detecting coronary artery disease in hypertensive patients: a meta-analysis. *European journal of nuclear medicine and molecular imaging.* 2011;38(11):2040-2049.

9. Guang Y, Xie L, Ding H, Cai A, Huang Y. Diagnosis value of focal liver lesions with SonoVue(R)-enhanced ultrasound compared with contrast-enhanced computed tomography and contrast-enhanced MRI: a meta-analysis. *Journal of cancer research and clinical oncology.* 2011;137(11):1595-1605.

10. Gurung A, Scrafford CG, Tielsch JM, Levine OS, Checkley W. Computerized lung sound analysis as diagnostic aid for the detection of abnormal lung sounds: a systematic review and meta-analysis. *Respir Med.* 2011;105(9):1396-1403.

11. Harnan SE, Cooper KL, Meng Y, et al. Magnetic resonance for assessment of axillary lymph node status in early breast cancer: a systematic review and meta-analysis. *European journal of surgical oncology : the journal of the European Society of Surgical Oncology and the British Association of Surgical Oncology.* 2011;37(11):928-936.

12. Kattenberg JH, Ochodo EA, Boer KR, Schallig HD, Mens PF, Leeflang MM. Systematic review and meta-analysis: rapid diagnostic tests versus placental histology, microscopy and PCR for malaria in pregnant women. *Malaria journal.* 2011;10:321.

13. Kocken M, Uijterwaal MH, de Vries AL, et al. High-risk human papillomavirus testing versus cytology in predicting post-treatment disease in women treated for high-grade cervical disease: a systematic review and meta-analysis. *Gynecologic oncology.* 2012;125(2):500-507.

14. Li R, Liu J, Xue H, Huang G. Diagnostic value of fecal tumor M2-pyruvate kinase for CRC screening: a systematic review and meta-analysis. *International journal of cancer. Journal international du cancer.* 2012;131(8):1837-1845.

15. Lin CY, Chen JH, Liang JA, Lin CC, Jeng LB, Kao CH. 18F-FDG PET or PET/CT for detecting extrahepatic metastases or recurrent hepatocellular carcinoma: a systematic review and meta-analysis. *European journal of radiology.* 2012;81(9):2417-2422.

16. Lucassen W, Geersing GJ, Erkens PM, et al. Clinical decision rules for excluding pulmonary embolism: a meta-analysis. *Annals of internal medicine.* 2011;155(7):448-460.

17. Lundstrom LH, Vester-Andersen M, Moller AM, et al. Poor prognostic value of the modified Mallampati score: a meta-analysis involving 177 088 patients. *British journal of anaesthesia.* 2011;107(5):659-667.

18. Mao R, Xiao YL, Gao X, et al. Fecal calprotectin in predicting relapse of inflammatory bowel diseases: a meta-analysis of prospective studies. *Inflammatory bowel diseases.* 2012;18(10):1894-1899.

19. Marton A, Xue X, Szilagyi A. Meta-analysis: the diagnostic accuracy of lactose breath hydrogen or lactose tolerance tests for predicting the North European lactase polymorphism C/T-13910. *Alimentary pharmacology & therapeutics.* 2012;35(4):429-440.

20. McInnes MD, Kielar AZ, Macdonald DB. Percutaneous image-guided biopsy of the spleen: systematic review and meta-analysis of the complication rate and diagnostic accuracy. *Radiology.* 2011;260(3):699-708.

21. Mugasa CM, Adams ER, Boer KR, et al. Diagnostic accuracy of molecular amplification tests for human African trypanosomiasis--systematic review. *PLoS neglected tropical diseases.* 2012;6(1):e1438.

22. Onishi A, Sugiyama D, Kogata Y, et al. Diagnostic accuracy of serum 1,3-beta-D-glucan for pneumocystis jiroveci pneumonia, invasive candidiasis, and invasive aspergillosis: systematic review and meta-analysis. *Journal of clinical microbiology.* 2012;50(1):7-15.

23. Pammi M, Flores A, Leeflang M, Versalovic J. Molecular assays in the diagnosis of neonatal sepsis: a systematic review and meta-analysis. *Pediatrics.* 2011;128(4):e973-985.

24. Papathanasiou ND, Boutsiadis A, Dickson J, Bomanji JB. Diagnostic accuracy of (1)(2)(3)I-FP-CIT (DaTSCAN) in dementia with Lewy bodies: a meta-analysis of published studies. *Parkinsonism Relat Disord.* 2012;18(3):225-229.

25. Phillips B, Wade R, Westwood M, Riley R, Sutton AJ. Systematic review and meta-analysis of the value of clinical features to exclude radiographic pneumonia in febrile neutropenic episodes in children and young people. *J Paediatr Child Health.* 2012;48(8):641-648.

26. Plana MN, Carreira C, Muriel A, et al. Magnetic resonance imaging in the preoperative assessment of patients with primary breast cancer: systematic review of diagnostic accuracy and meta-analysis. *European radiology.* 2012;22(1):26-38.

27. Sadigh G, Carlos RC, Neal CH, Dwamena BA. Ultrasonographic differentiation of malignant from benign breast lesions: a meta-analytic comparison of elasticity and BIRADS scoring. *Breast cancer research and treatment.* 2012;133(1):23-35.

28. Schmidt RL, Hall BJ, Layfield LJ. A systematic review and meta-analysis of the diagnostic accuracy of ultrasound-guided core needle biopsy for salivary gland lesions. *American journal of clinical pathology.* 2011;136(4):516-526.

29. Schmidt RL, Hunt JP, Hall BJ, Wilson AR, Layfield LJ. A systematic review and meta-analysis of the diagnostic accuracy of frozen section for parotid gland lesions. *American journal of clinical pathology.* 2011;136(5):729-738.

30. Smith TO, Back T, Toms AP, Hing CB. Diagnostic accuracy of ultrasound for rotator cuff tears in adults: a systematic review and meta-analysis. *Clinical radiology.* 2011;66(11):1036-1048.

31. Smith TO, Daniell H, Geere JA, Toms AP, Hing CB. The diagnostic accuracy of MRI for the detection of partial- and full-thickness rotator cuff tears in adults. *Magnetic resonance imaging.* 2012;30(3):336-346.

32. Summah H, Tao LL, Zhu YG, Jiang HN, Qu JM. Pleural fluid soluble triggering receptor expressed on myeloid cells-1 as a marker of bacterial infection: a meta-analysis. *BMC infectious diseases.* 2011;11:280.

33. Takakuwa KM, Keith SW, Estepa AT, Shofer FS. A meta-analysis of 64-section coronary CT angiography findings for predicting 30-day major adverse cardiac events in patients presenting with symptoms suggestive of acute coronary syndrome. *Academic radiology.* 2011;18(12):1522-1528.

34. Trallero-Araguas E, Rodrigo-Pendas JA, Selva-O'Callaghan A, et al. Usefulness of anti-p155 autoantibody for diagnosing cancer-associated dermatomyositis: a systematic review and meta-analysis. *Arthritis and rheumatism.* 2012;64(2):523-532.

35. Treglia G, Salsano M, Stefanelli A, Mattoli MV, Giordano A, Bonomo L. Diagnostic accuracy of (1)(8)F-FDG-PET and PET/CT in patients with Ewing sarcoma family tumours: a systematic review and a meta-analysis. *Skeletal radiology.* 2012;41(3):249-256.

36. Treglia G, Stefanelli A, Cason E, Cocciolillo F, Di Giuda D, Giordano A. Diagnostic performance of iodine-123-metaiodobenzylguanidine scintigraphy in differential diagnosis between Parkinson's disease and multiple-system atrophy: a systematic review and a meta-analysis. *Clinical neurology and neurosurgery.* 2011;113(10):823-829.

37. van Rijn RM, Wassenaar M, Verhagen AP, et al. Computed tomography for the diagnosis of lumbar spinal pathology in adult patients with low back pain or sciatica: a diagnostic systematic review. *European spine journal : official publication of the European Spine Society, the European Spinal Deformity Society, and the European Section of the Cervical Spine Research Society.* 2012;21(2):228-239.

38. Verhoeven CJ, Ruckert ME, Opmeer BC, Pajkrt E, Mol BW. Ultrasonographic fetal head position to predict mode of delivery: a systematic review and bivariate meta-analysis. *Ultrasound in obstetrics & gynecology : the official journal of the International Society of Ultrasound in Obstetrics and Gynecology.* 2012;40(1):9-13.

39. Wang GL, Zhao K, Liu ZF, Dong MJ, Yang SY. A meta-analysis of fluorodeoxyglucose-positron emission tomography versus scintigraphy in the evaluation of suspected osteomyelitis. *Nuclear medicine communications.* 2011;32(12):1134-1142.

40. Wang LW, Fahim MA, Hayen A, et al. Cardiac testing for coronary artery disease in potential kidney transplant recipients: a systematic review of test accuracy studies. *American journal of kidney diseases : the official journal of the National Kidney Foundation.* 2011;57(3):476-487.

41. Wang QB, Zhu H, Liu HL, Zhang B. Performance of magnetic resonance elastography and diffusion-weighted imaging for the staging of hepatic fibrosis: A meta-analysis. *Hepatology.* 2012;56(1):239-247.

42. Wang W, Li Y, Li H, et al. Immunodiagnostic efficacy of detection of Schistosoma japonicum human infections in China: a meta analysis. *Asian Pac J Trop Med.* 2012;5(1):15-23.

43. Wang Y, Zhang C, Liu J, Huang G. Is 18F-FDG PET accurate to predict neoadjuvant therapy response in breast cancer? A meta-analysis. *Breast cancer research and treatment.* 2012;131(2):357-369.

44. Wassenaar M, van Rijn RM, van Tulder MW, et al. Magnetic resonance imaging for diagnosing lumbar spinal pathology in adult patients with low back pain or sciatica: a diagnostic systematic review. *European spine journal : official publication of the European Spine Society, the European Spinal Deformity Society, and the European Section of the Cervical Spine Research Society.* 2012;21(2):220-227.

45. Xu HB, Li L, Xu Q. Tc-99m sestamibi scintimammography for the diagnosis of breast cancer: meta-analysis and meta-regression. *Nuclear medicine communications.* 2011;32(11):980-988.

46. Yu YH, Wei W, Liu JL. Diagnostic value of fine-needle aspiration biopsy for breast mass: a systematic review and meta-analysis. *BMC cancer.* 2012;12:41.

47. Zhang L, Zong ZY, Liu YB, Ye H, Lv XJ. PCR versus serology for diagnosing Mycoplasma pneumoniae infection: a systematic review & meta-analysis. *The Indian journal of medical research.* 2011;134:270-280.

48. Zhang Z, Lu B, Sheng X, Jin N. Accuracy of stroke volume variation in predicting fluid responsiveness: a systematic review and meta-analysis. *Journal of anesthesia.* 2011;25(6):904-916.

49. Zhou Y, Yin X, Ying J, Zhang B. Golgi protein 73 versus alpha-fetoprotein as a biomarker for hepatocellular carcinoma: a diagnostic meta-analysis. *BMC cancer.* 2012;12:17.
