# Supplementary material for: Time to publication among completed diagnostic accuracy studies: associated with reported accuracy estimates
Source: BMC Med Res Methodol. 2016 Jun 6;16:68. doi: 10.1186/s12874-016-0177-4 (PMC4896017; doi:10.1186/s12874-016-0177-4)
Supplement: Additional file 3: — Time from completion to submission: multivariable Cox regression analyses. (DOC 77 kb) [file 12874_2016_177_MOESM3_ESM.doc]

**Additional File 3:** Time from completion to submission: multivariable Cox regression analyses.

|  | | **Hazard ratio**  **(95%CI)^a^** | **p-value** |
| --- | --- | --- | --- |
| **Model 1: Sensitivity (n=327)** | |  |  |
| **Sensitivity (**logit transformed) | | 1.09 (1.04-1.15) | 0.001 |
| **Year of publication** (per 5 years) | | 0.84 (0.74-0.96) | 0.012 |
| **Journal impact factor** | |  |  |
|  | ≥4 | 1.14 (0.88-1.49) | 0.320 |
|  | <4 or not available | 1 |  |
| **Number of authors** | | 0.97 (0.93-1.00) | 0.084 |
| **Continent of first author** | |  |  |
|  | Europe, North America or Oceania | 0.61 (0.47-0.80) | <0.001 |
|  | Africa, Asia or South America | 1 |  |
| **Type of test** | |  |  |
|  | Imaging | 1.17 (0.87-1.57) | 0.300 |
|  | Other | 1 |  |
| **Type of data collection** | |  |  |
|  | Prospective | 1.16 (0.90-1.50) | 0.240 |
|  | Retrospective or not reported | 1 |  |
| **Study duration** (per year)^b^ | | 1.02 (0.98-1.07) | 0.320 |
| **Number of participants** (per 1000) | | 1.13 (0.94-1.35) | 0.190 |
| **Model 2: Specificity (n=324)** | |  |  |
| **Specificity** (logit transformed) | | 1.10 (1.04-1.17) | 0.001 |
| **Year of publication** (per 5 years) | | 0.88 (0.78-1.00) | 0.054 |
| **Journal impact factor** | |  |  |
|  | ≥4 | 1.23 (0.95-1.59) | 0.110 |
|  | <4 or not available | 1 |  |
| **Number of authors** | | 0.97 (0.93-1.00) | 0.067 |
| **Continent of first author** | |  |  |
|  | Europe, North America or Oceania | 0.65 (0.50-0.85) | 0.001 |
|  | Africa, Asia or South America | 1 |  |
| **Type of test** | |  |  |
|  | Imaging | 1.22 (0.94-1.59) | 0.140 |
|  | Other | 1 |  |
| **Type of data collection** | |  |  |
|  | Prospective | 1.16 (0.91-1.48) | 0.220 |
|  | Retrospective or not reported | 1 |  |
| **Study duration** (per year)^b^ | | 1.02 (0.97-1.06) | 0.440 |
| **Number of participants** (per 1000) | | 1.05 (0.88-1.26) | 0.570 |
| **Model 3: Youden’s index (n=322)** | |  |  |
| **Youden’s index** (logit transformed) | | 1.14 (1.06-1.22) | <0.001 |
| **Year of publication** (per 5 years) | | 0.87 (0.76-0.99) | 0.032 |
| **Journal impact factor** | |  |  |
|  | ≥4 | 1.17 (0.90-1.52) | 0.240 |
|  | <4 or not available | 1 |  |
| **Number of authors** | | 0.96 (0.92-1.00) | 0.034 |
| **Continent of first author** | |  |  |
|  | Europe, North America or Oceania | 0.63 (0.48-0.82) | <0.001 |
|  | Africa, Asia or South America | 1 |  |
| **Type of test** | |  |  |
|  | Imaging | 1.20 (0.91-1.59) | 0.190 |
|  | Other | 1 |  |
| **Type of data collection** | |  |  |
|  | Prospective | 1.20 (0.94-1.54) | 0.150 |
|  | Retrospective or not reported | 1 |  |
| **Study duration** (per year)^b^ | | 1.02 (0.97-1.06) | 0.500 |
| **Number of participants** (per 1000) | | 1.09 (0.91-1.30) | 0.350 |

^a^Frailty term added per meta-analysis to account for systematic differences in time from completion to publication between meta-analyses; variance of frailty terms were: model 1 = 0.097; model 2 = 0.042; model 3 = 0.067. ^b^One study was excluded from the Cox regression analysis because of a missing study duration.
