# Supplementary material for: Time to publication among completed diagnostic accuracy studies: associated with reported accuracy estimates
Source: BMC Med Res Methodol. 2016 Jun 6;16:68. doi: 10.1186/s12874-016-0177-4 (PMC4896017; doi:10.1186/s12874-016-0177-4)
Supplement: Additional file 4: — Time from submission to publication: multivariable Cox regression analyses. (DOC 77 kb) [file 12874_2016_177_MOESM4_ESM.doc]

**Additional File 4:** Time from submission to publication: multivariable Cox regression analyses.

|  | | **Hazard ratio**  **(95%CI)^a^** | **p-value** |
| --- | --- | --- | --- |
| **Model 1: Sensitivity (n=518)** | |  |  |
| **Sensitivity (**logit transformed) | | 0.98 (0.95-1.02) | 0.410 |
| **Year of publication** (per 5 years) | | 1.13 (1.03-1.25) | 0.012 |
| **Journal impact factor** | |  |  |
|  | ≥4 | 1.03 (0.85-1.25) | 0.780 |
|  | <4 or not available | 1 |  |
| **Number of authors** | | 1.03 (1.00-1.06) | 0.087 |
| **Continent of first author** | |  |  |
|  | Europe, North America or Oceania | 1.15 (0.95-1.41) | 0.160 |
|  | Africa, Asia or South America | 1 |  |
| **Type of test** | |  |  |
|  | Imaging | 0.94 (0.78-1.14) | 0.530 |
|  | Other | 1 |  |
| **Type of data collection** | |  |  |
|  | Prospective | 0.88 (0.73-1.05) | 0.150 |
|  | Retrospective or not reported | 1 |  |
| **Study duration** (per year)^b^ | | - | - |
| **Number of participants** (per 1000) | | 1.18 (1.01-1.38) | 0.035 |
| **Model 2: Specificity (n=514)** | |  |  |
| **Specificity** (logit transformed) | | 1.01 (0.97-1.05) | 0.670 |
| **Year of publication** (per 5 years) | | 1.13 (1.03-1.25) | 0.014 |
| **Journal impact factor** | |  |  |
|  | ≥4 | 1.03 (0.84-1.25) | 0.800 |
|  | <4 or not available | 1 |  |
| **Number of authors** | | 1.03 (1.00-1.06) | 0.066 |
| **Continent of first author** | |  |  |
|  | Europe, North America or Oceania | 1.15 (0.94-1.40) | 0.180 |
|  | Africa, Asia or South America | 1 |  |
| **Type of test** | |  |  |
|  | Imaging | 0.93 (0.77-1.12) | 0.420 |
|  | Other | 1 |  |
| **Type of data collection** | |  |  |
|  | Prospective | 0.88 (0.73-1.05) | 0.150 |
|  | Retrospective or not reported | 1 |  |
| **Study duration** (per year)^b^ | | - | - |
| **Number of participants** (per 1000) | | 1.20 (1.04-1.40) | 0.015 |
| **Model 3: Youden’s index (n=512)** | |  |  |
| **Youden’s index** (logit transformed) | | 0.96 (0.91-1.00) | 0.064 |
| **Year of publication** (per 5 years) | | 1.13 (1.02-1.24) | 0.015 |
| **Journal impact factor** | |  |  |
|  | ≥4 | 1.04 (0.86-1.26) | 0.700 |
|  | <4 or not available | 1 |  |
| **Number of authors** | | 1.03 (1.00-1.06) | 0.078 |
| **Continent of first author** | |  |  |
|  | Europe, North America or Oceania | 1.14 (0.93-1.39) | 0.200 |
|  | Africa, Asia or South America | 1 |  |
| **Type of test** | |  |  |
|  | Imaging | 0.94 (0.78-1.14) | 0.540 |
|  | Other | 1 |  |
| **Type of data collection** | |  |  |
|  | Prospective | 0.88 (0.73-1.05) | 0.150 |
|  | Retrospective or not reported | 1 |  |
| **Study duration** (per year)^b^ | | - | - |
| **Number of participants** (per 1000) | | 1.19 (1.02-1.38) | 0.029 |

^a^Frailty term added per meta-analysis to account for systematic differences in time from completion to publication between meta-analyses; variance of frailty terms were: model 1 = <0.001; model 2 = <0.001; model 3 = <0.001. ^b^Study duration excluded from this analysis due to missing data for >30%.
