# Supplementary material for: Time to publication among completed diagnostic accuracy studies: associated with reported accuracy estimates
Source: BMC Med Res Methodol. 2016 Jun 6;16:68. doi: 10.1186/s12874-016-0177-4 (PMC4896017; doi:10.1186/s12874-016-0177-4)
Supplement: Additional file 5: — Time to publication: sensitivity analyses. (DOC 62 kb) [file 12874_2016_177_MOESM5_ESM.doc]

**Additional File 5:** Time to publication: sensitivity analyses.

**Sensitivity analysis 1:** Excluding studies that reported the year, but not the month or exact date of completion of participant recruitment.

|  | | **Time from completion**  **to publication** | | | **Time from completion**  **to submission** | | |
| --- | --- | --- | --- | --- | --- | --- | --- |
|  | | **Studies**  **n (%)** | **Months**  **Median (IQR)** | **p-value** | **Studies**  **n (%)** | **Months**  **Median (IQR)** | **p-value** |
| **Overall** | | 418 (100) | 24 (16-35) |  | 297 (100) | 14 (7-24) |  |
| **Sensitivity^a,b^** | |  |  |  |  |  |  |
|  | <0.875 | 196 (47) | 25 (17-39) | 0.018 | 131 (44) | 16 (7-31) | 0.032 |
|  | ≥0.875 | 219 (52) | 23 (15-31) |  | 164 (55) | 13 (7-22) |  |
| **Specificity^a,c^** | |  |  |  |  |  |  |
|  | <0.899 | 194 (46) | 27 (18-38) | <0.001 | 140 (47) | 17 (9-30) | 0.001 |
|  | ≥0.899 | 217 (52) | 22 (15-31) |  | 152 (51) | 12 (6-22) |  |
| **Youden’s index^a,d^** | |  |  |  |  |  |  |
|  | <0.684 | 204 (49) | 27 (18-39) | <0.001 | 143 (48) | 17 (10-30) | <0.001 |
|  | ≥0.684 | 204 (49) | 22 (14-30) |  | 147 (49) | 11 (6-20) |  |

^a^Variables were dichotomized by a median-split. ^b^Sensitivity, ^c^Specificity, and ^d^Youden’s index missing for 7, 14 and 18 of 756 included studies, respectively.

**Sensitivity analysis 2:** Excluding studies that had not reported both a completion date of participant recruitment and a submission date.

|  | | **Time from completion**  **to publication** | | | **Time from completion**  **to submission** | | | **Time from submission**  **to publication** | | |
| --- | --- | --- | --- | --- | --- | --- | --- | --- | --- | --- |
|  | | **Studies**  **n (%)** | **Months**  **Median (IQR)** | **p-value** | **Studies**  **n (%)** | **Months**  **Median (IQR)** | **p-value** | **Studies**  **n (%)** | **Days**  **Median (IQR)** | **p-value** |
| **Overall** | | 330 (100) | 23 (16-34) |  | 330 (100) | 14 (7-25) |  | 330 (100) | 239 (181-333) |  |
| **Sensitivity^a,b^** | |  |  |  |  |  |  |  |  |  |
|  | <0.875 | 149 (45) | 24 (16-39) | 0.036 | 149 (45) | 16 (7-30) | 0.037 | 149 (45) | 235 (176-332) | 0.793 |
|  | ≥0.875 | 179 (54) | 21 (15-30) |  | 179 (54) | 13 (7-22) |  | 179 (54) | 239 (184-338) |  |
| **Specificity^a,c^** | |  |  |  |  |  |  |  |  |  |
|  | <0.899 | 152 (46) | 26 (17-38) | 0.002 | 152 (46) | 17 (9-30) | 0.001 | 152 (46) | 246 (184-333) | 0.479 |
|  | ≥0.899 | 173 (52) | 21 (15-31) |  | 173 (52) | 12 (6-22) |  | 173 (52) | 232 (176-333) |  |
| **Youden’s index^a,d^** | |  |  |  |  |  |  |  |  |  |
|  | <0.684 | 157 (48) | 25 (18-39) | <0.001 | 157 (48) | 17 (10-30) | <0.001 | 157 (48) | 245 (178-334) | 0.901 |
|  | ≥0.684 | 166 (50) | 21 (14-30) |  | 166 (50) | 11 (6-21) |  | 166 (50) | 233 (183-334) |  |

^a^Variables were dichotomized by a median-split. ^b^Sensitivity, ^c^Specificity, and ^d^Youden’s index missing for 7, 14 and 18 of 756 included studies, respectively.
